# Patient-Related Barriers to Digital Technology Adoption in Alzheimer Disease: Systematic Review

**DOI:** 10.2196/64324

**Published:** 2025-04-10

**Authors:** Andrea Panzavolta, Andrea Arighi, Emanuele Guido, Luigi Lavorgna, Francesco Di Lorenzo, Alessandra Dodich, Chiara Cerami

**Affiliations:** 1 IUSS Cognitive Neuroscience ICoN Center, Scuola Universitaria Superiore IUSS, Piazza della Vittoria, 15, Pavia, 27100, Italy, 39 3516237219; 2 Fondazione IRCCS Ca' Granda Ospedale Maggiore Policlinico, Milan, Italy; 3 Division of Neurology, Department of Advanced Medical and Surgical Sciences, AOU-University of Campania, Naples, Italy; 4 Experimental Neuropsychophisiology Unit, Fondazione Santa Lucia IRCCS, Rome, Italy; 5 Center for Mind/Brain Sciences-CIMeC, University of Trento, Rovereto, Italy; 6 Brain e-Health Aging (BeA) Laboratory, Department of Neurorehabilitation, Istituti Clinici Scientifici Maugeri IRCCS, Milan, Italy

**Keywords:** digital technology, digital e-health, accessibility, user-friendliness, neurocognitive disorders, Alzheimer disease, dementia

## Abstract

**Background:**

Digital technology in dementia is an area of great development with varying experiences across countries. However, novel digital solutions often lack a patient-oriented perspective, and several relevant barriers prevent their use in clinics.

**Objective:**

In this study, we reviewed the existing literature on knowledge, familiarity, and competence in using digital technology and on attitude and experiences with digital tools in Alzheimer disease. The main research question is whether digital competence and attitudes of patients and caregivers may affect the adoption of digital technology.

**Methods:**

Following the PRISMA guidelines, a literature search was conducted by two researchers in the group. Inter-rater reliability was calculated with Cohen κ statistics. The risk of bias assessment was also recorded.

**Results:**

Of 597 initial records, only 18 papers were considered eligible. Analyses of inter-rater reliability showed good agreement levels. Significant heterogeneity in study design, sample features, and measurement tools emerged across studies. Quality assessment showed a middle-high overall quality of evidence. The main factors affecting the adoption of digital technology in patients and caregivers are severity of cognitive deficits, timing of adoption, and the availability of training and support. Additional factors are age, type of digital device, and ease of use of the digital solution.

**Conclusions:**

Adoption of digital technology in dementia is hampered by many patient-related barriers. Improving digital competence in patient-caregiver dyads and implementing systematic, patient-oriented strategies for the development and use of digital tools are needed for a successful incorporation of digital technology in memory clinics.

## Introduction

The use of digital technology for the prevention, diagnosis, management, and assistance of patients with cognitive disorders and their caregivers has witnessed a dramatic increase in recent years. Telemedicine and eHealth technology services have proven to be valuable instruments for remote support and care in neurocognitive disorders, as consistently shown during the COVID-19 pandemic (see [1,2] for examples). The severe constraints on health care resources and the reduced time available to chronic patients during the pandemic have demonstrated the urgent need for the reorganization of memory clinic services to ensure adequate diagnosis and care [3]. Accordingly, many initiatives have tested digital technology solutions and telemedicine services in dementia settings [4,5], particularly in Alzheimer disease (AD), which represents one of the highest-risk and fastest-growing burdens on the health care system [6,7]. Literature evidence showed good levels of feasibility and effectiveness, particularly in individuals living in remote or underserved geographical areas or where in-person access to care facilities is limited [8]. Overall, patients with cognitive disorders and caregivers reported good satisfaction rates for digital technology, suggesting a general propensity toward adopting it routinely [8-10].

The literature highlights various solutions, including digital diagnostic instruments, tools for active and passive monitoring, and digital technologies supporting cognitive and motor rehabilitation. These tools have shown promising results not only in optimizing diagnostic pathways, disease management, and treatments, in avoiding dysfunctional and harmful disease trajectories, but also in helping the empowerment of patients and caregivers through active engagement in their care pathways [11,12]. However, despite the growing evidence suggesting the potential benefit of digital technology tools in cognitive patients, their integration into the daily routines of memory clinics is still limited [13].

Preliminary evidence suggested the presence of enabling factors such as familiarity, acceptability, and a positive attitude toward digital technology that could facilitate its adoption [9,13,14]. Conversely, inadequate experience with technology, poor digital literacy, and low education levels, as well as insufficient accommodation for motor and sensory impairments, may cause reduced acceptability and engagement in older adults [9,15]. However, a clear understanding of factors promoting or hampering the use of digital tools in memory clinics is still lacking.

Given these considerations, we reviewed existing literature with a particular interest in studies reporting features facilitating the use of digital technology in users (eg, competence, attitudes) or evaluating the performance of digital solutions (eg, usability). The main research question is whether digital competence and the attitudes of patients and caregivers may affect the adoption of digital technology. The final goal is to explore real-world needs, facilitators, and barriers in the field and to provide patient-oriented guidance for using and implementing novel digital technology tools in memory clinics.

## Methods

### Search Strategy and Selection Criteria

**Figure 1.**
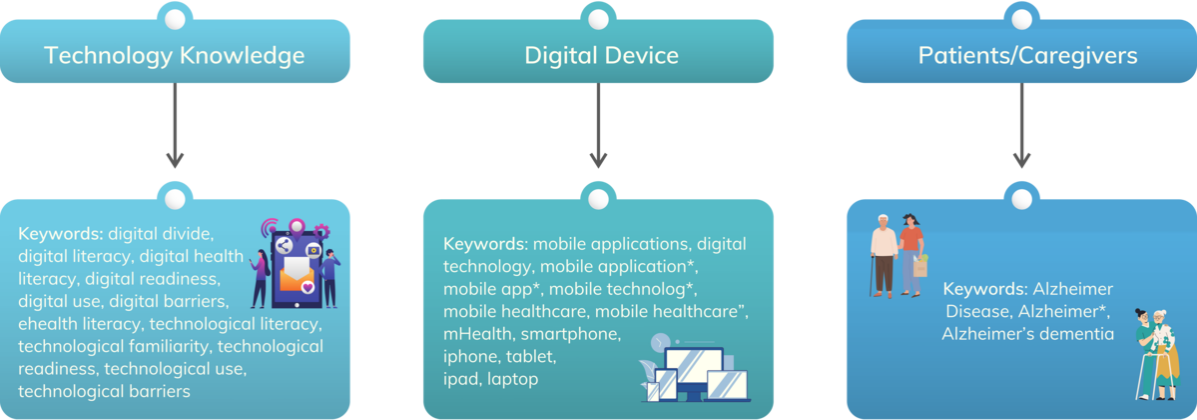
Keywords for the literature search.

A systematic search was conducted by two researchers of the group using PubMed, Scopus, Embase, and CINAHL (Cumulated Index in Nursing and Allied Health Literature) databases, following PRISMA (Preferred Reporting Items for Systematic Reviews and Meta-Analyses) guidelines [16]. According to our research hypothesis, the search strategy included a combination of MeSH (Medical Subject Headings) and relevant text terms focused on assessing familiarity and competence with digital technology and attitudes and experience regarding the use of digital solutions. See Figure 1 and Multimedia Appendix 1 for the full list of search terms. Given our theoretical framework and the growing body of literature on digital technologies in dementia, the search was focused on AD studies, aiming to improve consistency among findings.

Inclusion criteria for paper selection were as follows: (1) paper: full original research articles (excluding conference abstracts, case reports, reviews, and book chapters); (2) population of interest: individuals with AD (ranging from mild cognitive impairment to moderate-severe dementia stages) and their caregivers; (3) measurement: papers reporting quantitative and qualitative measures (ie, questionnaires, scales, or interviews) for assessing knowledge or familiarity with digital technology and attitude or experience in using digital solutions; (4) language and time span: papers published in English from 2010 up to October 15, 2023. Articles published before 2010 were excluded due to their reliance on outdated technologies, such as personal digital assistants, which may be less relevant for a contemporary audience.

### Study Selection and Data Extraction

The final set of records was uploaded onto Rayyan, a free web and mobile app designed to facilitate the initial screening of abstracts and titles through semiautomation, ensuring a high level of usability [17]. Data extraction was performed encompassing study identifiers (eg, authors, year of publication), sample features (eg, participants, diagnosis, group sizes), description of study methods (eg, type of digital solution and measurement), and findings. To ensure consistency in study selection and data extraction, the Cohen κ statistic was used to assess inter-rater reliability during the screening and eligibility phases [18]. Discrepancies were resolved through discussion, with input from an external senior expert when necessary.

### Risk of Bias Assessment

A risk of bias assessment was conducted using the Appraisal tool for Cross-Sectional Studies questionnaire [19] and the National Institutes of Health (NIH) Study Quality Assessment (SQA) Tool [20] for observational cohort and cross-sectional studies. These questionnaires evaluate the quality of human research studies, providing a global score as the rate of positive answers out of either 20 or 14 questions, and indicate overall quality and bias for each study. Considering the percentage of positive answers, categorical ratings were devised as follows: high quality (>70%), middle (70%‐50%), and low (≤49%) quality.

## Results

**Figure 2.**
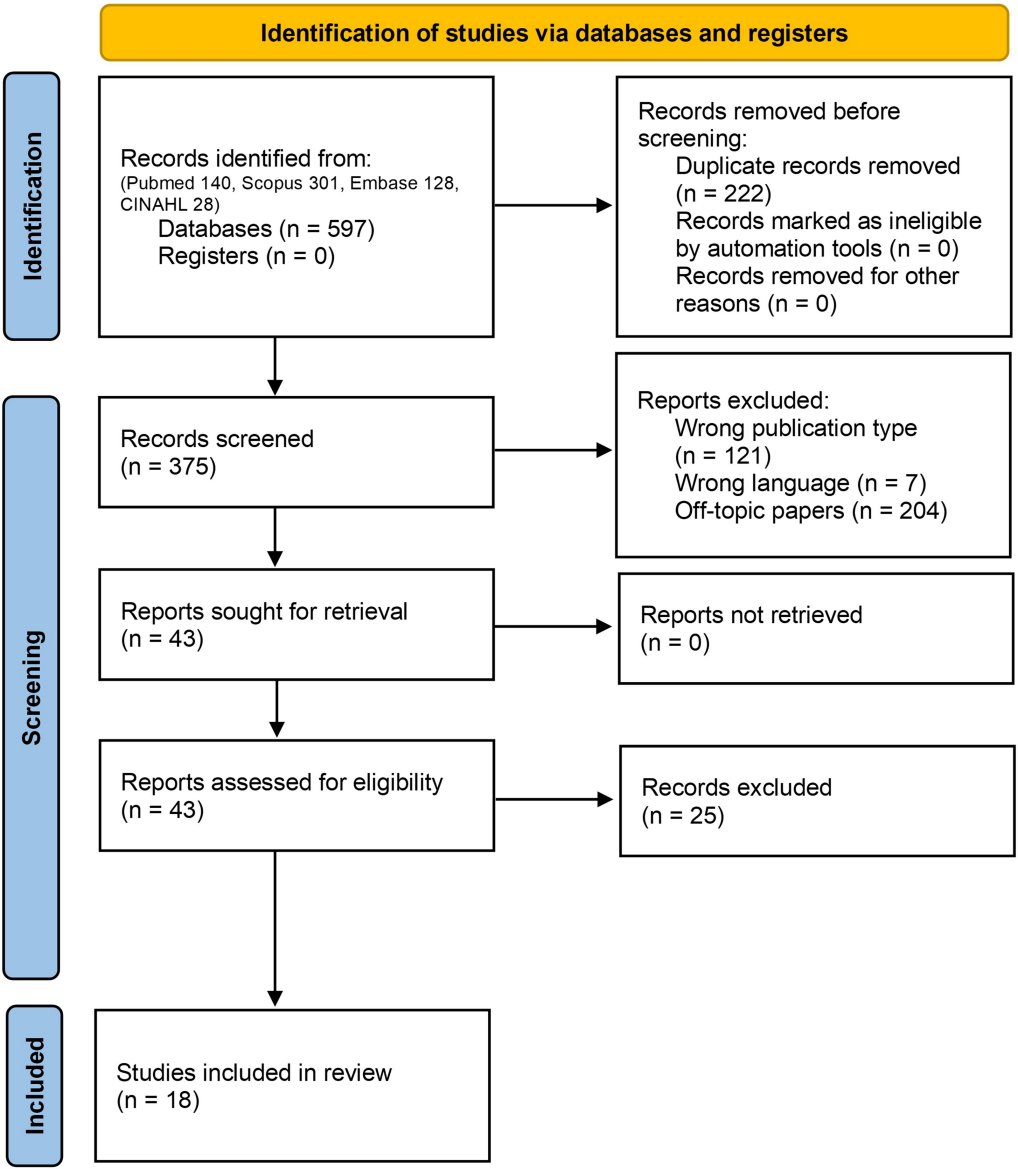
PRISMA flowchart reporting literature search strategy. CINAHL: Cumulated Index in Nursing and Allied Health Literature.

Out of the 597 articles initially retrieved, 554 papers were excluded during the screening phase (ie, 222 duplicates, 121 nonoriginal papers, 204 off-topic articles, and 7 articles not in English). A detailed review of the full texts led to the exclusion of 25 articles, with 18 papers meeting the eligibility criteria for this study. See Figure 2 for details of the PRISMA flow diagram. Analyses of inter-rater reliability showed good agreement levels for both the screening (κ=0.76; 94.46% agreement) and the eligibility (κ=0.75; 87.76% agreement) phases. Quality assessment of the included studies showed a middle-high quality of evidence.

Retained papers showed high heterogeneity in terms of study design (ie, qualitative vs quantitative), sample features (eg, patients and caregivers with various disease severity), and data type (eg, unstructured vs structured data). Given the high methodological and population-based heterogeneity across studies, results are presented as a narrative synthesis, focusing on shared themes and distinct sources of variability rather than on statistical aggregation.

See Tables1  2 for details of included papers and the quality assessment.

**Table 1.** Summary of papers exploring knowledge, familiarity, and competence with digital technology in patients with Alzheimer disease and caregivers.

Authors and country	Year	Sample	Measurements (qualitative/quantitative)	Study findings	Quality assessment
Guzman-Parra et al [21]; Spain, Sweden	2020	1086 MCI^a^ and dementia patient-caregiver dyads	Ad hoc-developed questionnaire to assess use and familiarity with touchscreen mHealth^b^ technology (TechPH questionnaire [22])	Low technophilia in patients, young, male, highly educated, living with children, having better mental health	High quality (NIH^c^ Study QA^d^ Tool Score=75%)
Jacobs et al [23]; US	2021	68 MCI patients 121 ADD^e^ patients	Ad hoc-developed web survey	Higher willingness to remote cognitive testing in MCI; significantly less access to video chat–capable technology in ADD; old age and low educational level influence access to technology and willingness to participate in remote cognitive assessment	High quality (NIH Study QA Tool Score=70%)
Christiansen et al [24]; Belgium, Spain, Sweden	2021	1082 MCI patients	Ad hoc-developed questionnaire to assess user experience with mHealth technology	Good to excellent QoL^f^ in participants with moderately or high technical skills; variation in technical skills and internet use was a relevant obstacle	High quality (NIH Study QA Tool Score=70%)
Wójcik et al [25]; Poland	2021	102 ADD caregivers	Ad hoc-developed questionnaires to assess acceptance of mHealth technology and laptop/PC use	Age, gender, and education level impact on technology acceptance; old age impact on computer but not on smartphone use; digital technology was perceived as useful for daily caring (locomotion, toileting, and meals)	High quality (NIH Study QA Tool Score=80%)
Albers et al [26]; US	2022	20 people with mild-to-moderate memory complaints patient-caregiver dyads	Semistructured interviews on digital technology use pre- and post-COVID-19 pandemic, adoption of digital technology during the pandemic, facilitators, and barriers to digital technology adoption	Technology use may benefit on maintaining social connections, alleviating boredom, and fostering caregiver relief; patient dependence and low technological literacy prevented the use of digital technology	High quality (NIH Study QA Tool Score=70%)
Talbot and Briggs [27]; UK	2022	19 ADD, mixed and related dementia patients	Semistructured interviews on digital technology use and experience during the COVID-19 pandemic	Low usability and cognitive fatigue in the use of digital technologies; need for training on the use and for a better engagement	Middle quality (NIH Study QA Tool Score=60%)
Nguyen et al [28]; US	2022	124 ADD and related dementia caregivers	Ad hoc-developed web survey to assess experience with iN2L Samsung Galaxy Tablet	Benefit on caregiver wellbeing, alleviating stress, increasing satisfaction, and improving access to supportive programs	Middle quality (NIH Study QA Tool Score=64%)
Wilson et al [29]; UK	2023	29 MCI, ADD and related dementia patient-caregiver dyads	Videoconferencing or phone interviews of m-health technology use	Smart devices are valuable, versatile tools for essential and meaningful activities, and necessary devices to participate in modern life; need for training on the use of smart devices	Middle quality (NIH Study QA Tool Score=50%)

a MCI: Mild cognitive impairment.

b mHealth: mobile health.

c NIH: National Institutes of Health.

d QA: Quality assessment.

e ADD: Alzheimer disease dementia.

f QoL: Quality of Life.

**Table 2.** Summary of papers exploring use, acceptability, and usability of digital solutions in AD patients and caregivers.

Authors and country	Year	Sample	Digital solution	Measurements (qualitative/quantitative)		Study findings	Quality assessment
Lim et al [Bibr R30][30]; Australia	2013	21 unspecified dementia patient-caregiver dyads	iPad and 11 iPad apps	Ad hoc-developed questionnaires to assess experience, ability to use, engagement and utility		Half of dementia patients were able to engage with and use the iPad independently	Middle quality (NIH^a^ Study QA^b^ Tool Score=60%)
Zmily et al [31][Bibr R31]; Jordan	2014	10 early ADD^c^ patients	Samsung Galaxy Tablet and Android app (ADcope)	Ad hoc-developed questionnaires, NASA-TLX^d^ index workload assessment [32]		Low workload scores; good post-task satisfaction; successful use even without any prior experience	Middle quality (NIH Study QA Tool Score=50%)
Brown et al [33][Bibr R33]; US	2016	11 ADD caregivers	Android app (CareHeroes App)	Ad hoc-developed web-based survey		Perceived easiness to perform tasks despite medium-low proficiency with technology	High quality (NIH Study QA Tool Score=80%)
Killin et al [34][Bibr R34]; UK	2018	10 ADD, vascular or mixed dementia patient-caregiver dyads	Digital Support Platform (DSP)	Semistructured interviews		Caregiver use was better than that of patients; High interest in learning to use technology more effectively and enjoyed having their own tablet devices; Need of training in the use of new technology	Middle quality (NIH Study QA Tool Score=50%)
Ruggiano et al [35][Bibr R35]; US	2019	36 ADD and related dementia caregivers	Android app (Care IT)	Interviews and focus group		eHealth and individual technologies may not fully meet the needs of caregivers; need for more effective, easy-to-use, and more widely disseminated – especially for caregivers from a disadvantaged background	High quality (NIH Study QA Tool Score=70%)
Øksnebjerg et al [36][Bibr R36]; Denmark	2020	112 ADD, vascular dementia patients 98 caregivers	ReACT (Rehabilitation in Alzheimer disease using Cognitive support Technology) app	Ad hoc-developed web-based survey (including USEdem questionnaire)		Need for timely introduction of digital technology; need of caregiver support for the adoption of digital solution	High quality (NIH Study QA Tool Score=85%)
Evans et al [37][Bibr R37]; UK	2021	26 ADD and related dementia patient-caregiver dyads	App-based prompter for a touchscreen Tablet	Semistructured interviews on experience and usefulness		Attitudes to technology, perceived utility, and emotional impact of needing help impact the acceptance	High quality (NIH Study QA Tool Score=80%)
Berge et al [38][Bibr R38]; Norway	2022	24 ADD patient-caregiver dyads	iOS app (Alight)	SUS^e^ scale [39] modified; ad hoc-developed questionnaire to explore adoption, user-friendliness, usefulness, and impact		High adoption and feasibility; Need of timely introduction; 50% of the accepting dyads had difficulties independently managing the digital solution	Middle quality (NIH Study QA Tool Score=65%)
Skirrow et al [40][Bibr R40]; UK, US	2022	73 MCI/mild^f^ ADD patients 78 healthy control subjects	Story recall task app	Ad hoc-developed questionnaires to assess usability and task engagement		Technical problems, easy use of the app, and a broad interest in the tasks; Modest improvement of recall	High quality (NIH Study QA Tool Score=79%)
Rossetto et al [41][Bibr R41]; Italy	2023	11 MCI, 19 mild AD patient-caregiver dyads	Telerehabilitation app and web-based software (ABILITY)	SUS scale [39], percentage of sessions attended within 6 weeks (adherence)		High adherence rate, usability, and treatment efficacy on global cognitive level in the digital-treated group	High quality (NIH Study QA Tool Score=93%)

a NIH: National Institutes of Health.

b QA: Quality assessment.

c ADD: Alzheimer disease dementia.

d NASA TLX: NASA Task load index.

e SUS: System usability scale.

f MCI: Mild cognitive impairment.

### Evidence From Papers Reporting Knowledge, Familiarity, and Competence With Digital Technology

Among studies investigating factors affecting knowledge, familiarity, and competence in digital mobile health (mHealth) technology use [21,23-29], the degree of cognitive impairment was the first factor significantly affecting digital competence in the adoption of technology solutions. Individuals with milder cognitive deficits showed higher competence in the use of digital devices and the internet and consequently, greater willingness levels to participate in studies compared to those with more advanced cognitive decline [23,24]. Overall, users exhibited greater openness to digital technology when they had prior experience and familiarity and good digital literacy [21,23,24]. Significant challenges were reported in studies related to limited technical skills of both patients and caregivers [26-28]. Nonetheless, while overall caregivers showed greater competence and enthusiasm for digital technology than patients, other factors, as male sex, higher education, and younger age, impact at the individual level on familiarity and willingness to participate to studies [21,25].

Whereas smartphone acceptance is consistent across age groups, computer use declined with age and in patients versus caregivers, indicating an age-related barrier to some technologies [27-29]. For example, the use of tablets represented a significant barrier in the VITAL at Home project [28], in which caregivers required substantial guidance to effectively use the software implemented in tablets.

### Evidence From Papers Reporting Acceptability and Usability of Digital Solutions

Studies examining the use of digital solutions among AD patients and caregivers [30,31,33-38,40,41] consistently found that IT system accessibility and ease of use of the proposed solution are crucial factors influencing adoption rates. Ease of use of the digital health solutions and attitude to technology represent key factors enhancing acceptance of digital tools in daily routines [30,31,33,35,37,40]. The likelihood of sustained use is related to the timing of their adoption within the disease course, with earlier implementation correlating with better long-term engagement for both patients and caregivers [34,36]. The provision of informal support and training is another enabling factor ensuring long-term and better use [31,36,41]. As the disease progresses, the severity of cognitive decline and the perceived cognitive workload pose relevant challenges for constant engagement and use [31,37,38].

## Discussion

Digital technology in dementia is an area of rapid development marked by diverse approaches across countries. A huge step forward has been made during the COVID-19 pandemic, acting as a catalyst for extensive research and development of eHealth and mHealth solutions, favoring the testing of digital solutions in memory clinics and promoting their implementation in practice [2,4,42]. In this study, we reviewed the existing literature on digital competence, attitude to technology, and use of digital tools in AD, with the final aim to underline main factors affecting the adoption of digital solutions and provide useful information for their future development and use in memory clinics.

Literature evidence revealed several barriers in adopting digital technology in dementia. Among the main factors, the severity of cognitive deficits is the first key element. As adherence to digital technology interventions decreases with the severity of cognitive decline [23,24,31,37,38], clinicians and researchers should embrace technology-based solutions at an earlier stage of the care pathway with the aim to improve use and effectiveness. With the progression of the disease, poor verbal and motor initiative may affect patient engagement with digital technology, reducing interest, initiative, and participation [27]. In such cases, behavioral or sensory-based interventions, such as sensory stimulation delivered through digital devices, represent an appropriate choice. Therefore, an in-depth assessment of the behavioral and cognitive profile should always guide the specialist to choose the right digital tool to use and address patients accordingly.

Age, digital literacy, and special needs (eg, visual or auditory impairments) are additional variables that limit the access or use of digital technology both for patients and caregivers, without affecting the will to participate in telecare services [21,25,43]. These aspects are of particular relevance in memory clinics, where final users are often patients and caregivers in old adulthood [44]. The type of digital device may also impact the feasibility and real-life implementation of digital protocols, with smartphones being more accessible compared to computers or other devices [27,29,30]. Consequently, smartphones show higher acceptability scores in patients [45,46], although some challenges in the use of touchscreen and software updates are reported, particularly among those with poor digital literacy and visual impairments [24].

Another crucial barrier to the stable use of eHealth and mHealth solutions is the need for training and support [27,29,30,34,36]. The availability of formal support in the technology setup or training has been shown to positively impact willingness and acceptability [30,34,41]. As individuals may be unlikely to embrace burdensome technologies [26], preliminary training at health care facilities before the in-home adoption should be recommended to overcome potential technophobia in patients and caregivers [13,21]. The evaluation of specific digital needs in each single patient-caregiver dyad may guarantee a more inclusive and practical approach.

Therefore, the lack of active involvement of final users in the design, development, and testing of novel digital solutions crucially contributes to the poor applicability of these instruments in real-life scenarios [47].

In addition, developing technologies without user burden is pivotal for their success. Minimizing the burden on caregivers is of paramount importance, as they are often responsible for managing digital tools (eg, calendars for medication or televisits). The mitigation of stress and anxiety due to the use of technologies is crucial for caregivers [26,35,37,38], particularly for those individuals with disadvantageous socioeconomical backgrounds, who face additional challenges in adapting to the tools and using them effectively [35]. Co-design with end-users and adaptation of the digital tool to real-life needs are therefore critical [47].

Several limitations characterize the body of research analyzed in this review. Many studies involved small sample sizes that were not fully representative of the AD population, often excluding individuals who were unable or unwilling to participate for various reasons. Availability of high-speed internet connection [23,24], recruitment or screening modality (eg, through email, social media or videoconference) [26,27], and socioeconomic status of participants [23,37] are additional variables affecting results, while study design (eg, lack of control group or randomized approach [28]) and measurement issues (eg, lack of pre-post measures [36]) are crucial aspects that hamper the reliability of data findings.

In conclusion, digital tools have the potential to significantly influence the quality of dementia services acting on different dimensions [13]. They can increase effectiveness by enabling faster access to specialist care, better diagnoses and treatments, and preventing avoidable hospitalizations. They improve timeliness, reducing waiting lists and unnecessary travel. They ensure patient-centeredness and safe care, providing care tailored to individual needs and values and treating patients more appropriately. They provide integrated, more efficient, and equitable care by taking an interdisciplinary approach involving multiple specialists, improving cost-effectiveness, and overcoming geographic barriers addressing cultural diversity. However, the adoption of digital technology is limited by many patient-related barriers. Improving digital competence in the patient-caregiver dyads and implementing a systematic patient-oriented strategy for the development and use of digital tools (eg, by promoting participated design, early timing of solution adoption and availability of training and technical support) remain critical factors to consider for the successful incorporation of digital eHealth and mHealth solutions and services into future memory clinics.

## Supplementary material

10.2196/64324 Multimedia Appendix 1 Combination of Medial Subject Headings (MeSH) and text words used for literature search in different databases.

10.2196/64324 Checklist 1 PRISMA 2020 checklist for reporting systematic reviews and meta-analyses.

## Abbreviations

AD: Alzheimer disease
CINAHL: Cumulated Index in Nursing and Allied Health Literature
NIH: National Institutes of Health
PRISMA: Preferred Reporting Items for Systematic Reviews and Meta-Analyses
SQA: Study Quality Assessment
