# Supplementary material for: Patient-Related Barriers to Digital Technology Adoption in Alzheimer Disease: Systematic Review
Source: JMIR Aging. 2025 Apr 10;8:e64324. doi: 10.2196/64324 (PMC12005595; doi:10.2196/64324)
Supplement: Multimedia Appendix 1 [file aging-v8-e64324-s001.docx]

**Supplementary file 1.** Combination of Medial Subject Headings (MeSH) and text words used for the literature search in different database

**Pubmed**

**Concept 1: Technology Knowledge**

**Keywords:** “digital divide”[Mesh] OR “digital literacy”[tw] OR “digital health literacy”[tw] OR “digital readiness”[tw] OR “digital use”[tw] OR “digital barriers”[tw] OR “ehealth literacy”[tw] OR “technological literacy”[tw]  OR “technological familiarity”[tw] OR “technological readiness”[tw] OR “technological use”[tw] OR “technological barriers”[tw] OR technolog*[tw]

**Concept 2: Digital Device**

**Keywords:** “mobile applications”[Mesh] OR "digital technology"[Mesh] OR “mobile application*”[tw] OR “mobile app*”[tw] OR “mobile technolog*”[tw] OR “mobile healthcare”[tw] OR “mobile healthcare”[tw] OR mHealth[tw] OR smartphone[tw] OR iphone[tw] OR tablet[tw] OR ipad[tw] OR laptop[tw]

**Concept 3: Patients/caregivers**

**Keywords:** "Alzheimer Disease"[Mesh] OR Alzheimer*[tw] OR "Alzheimer’s dementia"[tw]

**Scopus**

**Concept 1: Technology Knowledge**

**Keywords:** “digital divide” OR “digital literacy” OR “digital health literacy” OR “digital readiness” OR “digital use” OR “digital barriers” OR “ehealth literacy” OR “technological literacy” OR “technological familiarity” OR “technological readiness” OR “technological use” OR “technological barriers” OR technolog*

**Concept 2: Digital Device**

**Keywords:** “mobile applications” OR "digital technology" OR “mobile application*” OR “mobile app*” OR “mobile technolog*” OR “mobile healthcare” OR “mobile healthcare” OR mHealth OR smartphone OR iphone OR tablet OR ipad OR laptop

**Concept 3: Patients/caregivers**

**Keywords:** "Alzheimer Disease" OR Alzheimer* OR "Alzheimer’s dementia"

**Embase**

**Concept 1: Technology Knowledge**

**Keywords:** ‘digital divide’:ti,ab,kw OR ‘digital literacy’:ti,ab,kw OR ‘digital health literacy’:ti,ab,kw OR ‘digital readiness’:ti,ab,kw OR ‘digital use’:ti,ab,kw OR ‘digital barriers’:ti,ab,kw OR ‘ehealth literacy’:ti,ab,kw OR ‘technological literacy’:ti,ab,kw OR ‘technological familiarity’:ti,ab,kw OR ‘technological readiness’:ti,ab,kw OR ‘technological use’:ti,ab,kw OR ‘technological barriers’:ti,ab,kw OR ‘technology’:ti,ab,kw

**Concept 2: Digital Device**

**Keywords:** ‘mobile applications’:ti,ab,kw OR ‘digital technology’:ti,ab,kw OR ‘mobile app’:ti,ab,kw OR ‘mobile technology’:ti,ab,kw OR ‘mobile healthcare’:ti,ab,kw OR ‘mobile healthcare’:ti,ab,kw OR ‘mHealth’:ti,ab,kw OR ‘smartphone’:ti,ab,kw OR ‘iphone’:ti,ab,kw OR ‘tablet’:ti,ab,kw OR ‘ipad’:ti,ab,kw OR ‘laptop’:ti,ab,kw

**Concept 3: Patients/caregivers**

**Keywords:** ‘Alzheimer Disease’/de OR ‘Alzheimer’:ti,ab,kw OR ‘Alzheimer dementia’/de

**CINAHL**

**Concept 1: Technology Knowledge**

**Keywords:** MH “digital divide” OR “digital literacy” OR “digital health literacy” OR “digital readiness” OR “digital use” OR “digital barriers” OR “ehealth literacy” OR “technological literacy” OR “technological familiarity” OR “technological readiness” OR “technological use” OR “technological barriers” OR “technology”

**Concept 2: Digital Device**

**Keywords:** MH “mobile applications” OR MH “digital technology” OR “mobile application” OR “mobile app” OR “mobile technology” OR “mobile healthcare” OR “mobile healthcare” OR “mHealth” OR “smartphone” OR “iphone” OR “tablet” OR “ipad” OR “laptop”

**Concept 3: Patients/caregivers**

**Keywords:** MH “Alzheimer disease” OR “Alzheimer” OR “Alzheimer dementia”
